# Switchable Multi-Color Solution-Processed QD-laser

**DOI:** 10.1038/s41598-020-60859-w

**Published:** 2020-03-24

**Authors:** Samiye Matloub, Pegah Amini, Ali Rostami

**Affiliations:** 1 Quantum Photonics Research Lab (QPRL), University of Tabriz, Tabriz, 5166614761 Iran; 2 Photonics and Nanocrystals Research Lab (PNRL), University of Tabriz, Tabriz, 5166614761 Iran; 3 SP-EPT Lab., ASEPE Company, Industrial Park of Advanced Technologies, Tabriz, 5364196795 Iran

**Keywords:** Diode lasers, Semiconductor lasers

## Abstract

In this paper, for the first time, the switchable two-color quantum dot laser has been realized considering solution process technology, which has both simultaneous and lonely lasing capability exploiting selective energy contacts. Furthermore, both channels can be modulated independently, which is a significant feature in high-speed data transmission. To this end, utilizing superimposed quantum dots with various radii in the active layer provides the different emission wavelengths. In order to achieve the different sizes of QDs, solution process technology has been used as a cost-effectiveness and fabrication ease method. Moreover, at the introduced structure to accomplish the idea, the quantum wells are used as separate selective energy contacts to control the lasing channels at the desired wavelength. It makes the prominent device have simultaneous lasing at different emission wavelengths or be able to lase just at one wavelength. The performance of the proposed device has been modeled based on developed rate equation by assuming inhomogeneous broadening of energy levels as a consequence of the size distribution of quantum dots and considering tunnel injection of carriers into the quantum dots via selective energy contacts. Based on simulation results, the simultaneous lasing in both or at one of two wavelengths 1.31  μm and 1.55  μm has been realized by the superimposition of two different sizes of InGaAs quantum dots in a single cavity and accomplishment of selective energy contacts. Besides, controlling the quantum dot coverage leads to managing the output power and modulation response at the desired wavelengths. By offering this idea, one more step is actually taken to approach the switchable QD-laser by the simple solution process method.

## Introduction

Nowadays, multi-wavelength lasers have been committed to numerous applications in the field of imaging, tomography and ultra-fast data communication. Thus, the ability to achieve simultaneous lasing at different wavelengths has been the subject of enormous research in the past few years^1–5^. In addition, lasers have drawn great attention due to their wide potential applications in all-optical switching system^6^ and on/off switching behavior^7^, ultrafast photonics^8–10^, playing roles as the light irradiation source for cancer therapy^11–14^, analyzing the photo-thermal properties^15–17^ and excitation source for Raman spectra measurement^18,19^. Moreover, the high performance of lasers can be accomplished by utilizing quantum dots (QDs) in the active region of laser diodes. Hence, the quantum dot lasers (QD-Lasers) have been received great attention in recent decades among different types of laser structures^20–25^. The low threshold current, high modulation bandwidth, narrow linewidth, low-frequency chirp, and temperature stability are the superior advantages of QD-lasers due to the QDs’ unique properties such as quantum confinement effect and delta-function-like density of states^4,26–29^. Consequently, the realization of switchable multi-wavelength lasers based on QD-lasers has been promising high-speed data transmissions in the telecommunications world.

The solution process is cost-effectiveness and straightforward chemical method for synthesis QDs. Recently, many optoelectronic devices like QD-SOA, QD-LED, Luminescent Solar Concentrator, and QD-Infrared photodetector have been implemented utilizing solution-processed QDs^30–34^. In the solution process technology, the tunable size of QDs can be achieved by exploiting the simple chemical procedures. Basically, the size of QDs can be controlled by providing suitable experimental conditions such as the concentration of materials, temperature, pH and the speed of rotation of solvent^35,36^. In this technology, the diameter range of QDs can be tuned from 1 nm to 10 nm^30,31,37^. However, having the 0.1 nm size deviation for synthesized QD in the appropriate experimental condition is possible^37–39^. In better words, the different sizes of QD groups in the active region of laser diodes can be easily implemented based on the solution process method, leading to the realization of multi-wavelength QD-Laser^40^.In the proposed multi-wavelength QD-Laser, the injected carriers diffuse in separated confinement heterostructure (SCH) layer and then relax into all QD groups. So, all of them start lasing simultaneously^40^.

Controlling both the separate lasing and simultaneous lasing is accomplished by utilizing the concept of electron path channelization. The carriers injected to the SCH layer can be channeled into the specific QD groups in the active region, as a result of tunneling injection^41–46^ of carriers into each group of QDs via tunneling from separate selective energy contacts (SECs) between SCH and optical confinement layer of multi-wavelength QD-laser. In the proposed multi-color QD-laser by utilizing SECs, carriers with specific energy can be directly injected into the ground level of energy in selected QDs, leading to simultaneously or individually lasing of each QD groups.

**Figure 1:**
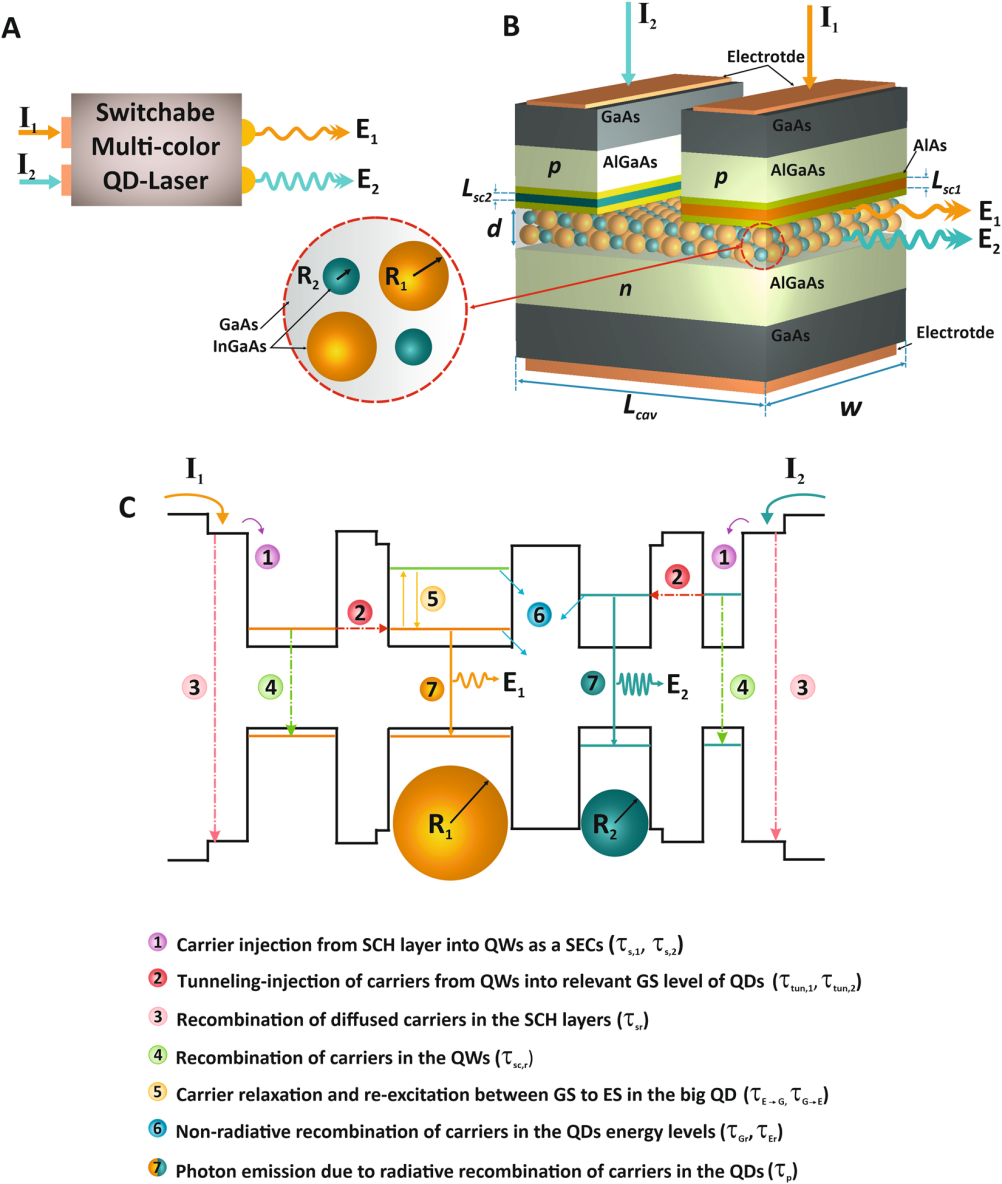
The schematic and performance of the proposed switchable two-color QD-laser utilizing SECs. (**A**) The block diagram of the proposed switchable two-color QD-laser. (**B**) The structure of two-color QD-laser with superimposing two different sizes of InGaAs QDs in active region and two SEC corresponding to each QD groups located between SCH layer and active region to tunnel injection of carriers directly into the related QD groups with the same energy levels. (**C**) The energy band diagram of the proposed structure including the carrier relaxation process into QDs.

In this paper, the switchable two-color QD-laser exploiting SECs have been introduced and designed for the first time. The aim of this proposed model is achieving to controllable lasing either simultaneously or singly at different emission wavelengths by SECs which can be useful in different applications such as laser gyroscope^47^, optical spectroscopy and dense wavelength division multiplexing (DWDM)^48^. In this device, illustrated in Fig. 1(A), achieving the simultaneous or individually emission wavelengths in 1.31 μm and 1.55 μm can be possible by utilizing two different sizes of QDs in the active region. Both of the 1.31 μm and 1.55 μm are prevalent wavelengths for optical communication applications^5,49–51^; thus, the investigations of this study are carried out in the emission regions that are determined by QDs’ sizes. Moreover, two SECs are used each of which injected carriers into the related QDs via tunneling effects. Hence, both of the output emission can be modulated independently, which is a significant feature in high-speed data transmission. The SECs has been designed in a way that their energy levels are proportionate with the ground state (GS) of QDs. Therefore, the carriers directly injected into the GS of QDs, according to the injection of carriers in the relevant SECs, make them start lasing simultaneously or individually.

Last but not least, the proposed switchable two-color QD-laser can be easily implemented by solution process technology, which provides the low-cost and simple chemical system with controllable experimental conditions and permits us to synthesis tunable colloidal QDs, nevertheless, due to some insignificant limitation of synthesis conditions, attaining the exact size of QDs is limited. Therefore, the size distribution of each QD group leads to broadening in energy levels of QD-laser structure, which inhomogeneously broadening (IHB) of energy levels has to be considered modeling the performance of QD-laser in developed rate equation framework from the practical point of view^20,52,53^.

## The proposed switchable two-color QD-laser using SECs

As stated above, in this paper the switchable two-color solution-processed QD-laser exploiting SECs has been proposed by the ability to choose lasing wavelengths. In this QD-laser, the quantum well (QW)^44,54^ is used as the SEC to control the lasing channels. Here, the most usual 1.31 μm and 1.55 μm wavelengths in the telecommunication applications are considered. As seen in the schematic view of the device structure in Fig. 1(B), for the realization of two-color lasing, two different sizes of InGaAs/GaAs QDs with radiuses of R_1_ and R_2_ are superimposed in the active region of QD-laser, so the lasing from two independent channels becomes possible. For facile access to this structure, the simple and low-cost solution process method is suggested, which is called “wet,” and it provides the chemical system with controllable experimental conditions. The synthesis of colloidal QDs by chemical procedures in solution allows achieving tunable sizes of QDs^30,55^; also the creation of suitable conditions for synthesis in this method is more convenient. The solution process is economical compared with the other expensive methods like epitaxial which needs a high-vacuum condition^30,31^.

Injection of carriers into selected groups of QDs can be realized by SECs. Thus, the width of the QW is chosen as their energy state is the same with the GS energy of relevant QDs; then tunneling injection of carriers occurs via the barriers of QW into QDs, so they get localized in them. This process causes the fast transition of carriers^30^ and improves the performance of QD-laser. Figure 1(B) exhibits the two-color QD-laser structure in which the active layer of QD-laser includes two different sizes of InGaAs QDs that is sandwiched by p and n-type AlGaAs as a carrier transition layer. The p and n-type GaAs are cladding layers. Also, the QW contacts contain the InGaAs well and AlAs barriers. As seen in the figure, current injection in each of the contacts can active them, therefore if the current is injected in both of the contacts, the output emission occurs in two colors, but the injection of current just in one contact leads to having one color emission; note that the key idea is the operation of SECs to control the output emissions, accordingly the title of switchable two-color QD-laser by selective contact can be chosen for this structure. The process of the system starts with the diffusion of carriers in the SCH layer by current injection after that the carriers relax into the QW SECs. The collection of carriers, tunnel from QW state into GS of QD, and then the transmitted carriers recombine radiatively or non-radiatively.

Figure 1(C) illustrates the carriers act in the energy band diagram of switchable two-color QD-laser by SEC. Obviously, the big QD that determines the channel1, has both GS and excited state (ES), whereas the small QD relates to channel2 with only GS. The carriers and photons dynamics like capture and relaxation in the QD describes the performance of QD-lasers. As it is shown in detail in the figure, at first, the diffusion of the carriers occurs in the SCH layer (τ_si_) and then some of those carriers tunnels into the QDs (τ_tun,i_). These carriers recombine radiatively or non-radiatively; the lifetime constant of recombination of diffused carriers in the SCH layer and in SECs are τ_sr_ and τ_sc,r_, respectively. In the big QDs, the carrier relaxed from ES to GS by the rate of τ_E→G_; also some of the carriers re-excited from ES to GS by the rate of τ_G→E_. Moreover, the non-radiative recombination of carriers in the QDs energy levels determine by τ_Er_ and τ_Gr_. Finally, the stimulated emission leads to the photon emission from the ground state by the rate of τ_p_.

## Concept and Theoretical Modeling

**Figure 2:**
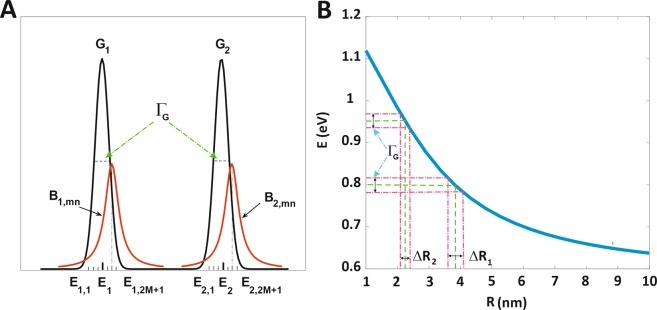
The IHB and HB of energy levels for two groups of QDs relevant to each emission channel. (**A**) The black solid indicates superimposition of two Gaussian profiles for two groups of QDs. The FWHM of each Gaussian profiles equals to Γ_G_; also the HB is included in the diagram (red curves). (**B**) The E-R diagram demonstrates energy of emission wavelengths as a function of QD radii (The minimum and maximum values of E are 0.8 eV for R_1_ = 3.85 nm and 0.946 eV for R_2_ = 2.47 nm respectively relates to 1.55  μm and 1.31  μm emission wavelengths). ∆R_1_ and ∆R_2_ show size distribution over both specific R_1_ and R_2_ appropriate to FWHM of Gaussian profiles; i.e. Γ_G_.

In this section, the theoretical model is provided for the proposed structure. As mentioned before, the aim of this paper is achieving to a switchable two-color QD-laser by selective contacts. According to reports, some of the research groups have suggested the InGaAs QDs for QD-laser structure^20,23,56^. Therefore, utilizing the two QDs of InGaAs/GaAs with radiuses of 2.47 nm and 3.85 nm in the active region leads to achieving two colors at an independent emission wavelength of 1.31 μm and 1.55 μm, respectively. Even though the solution process is a low-cost method which provides a simple chemical system to achieve controllable experimental condition to synthesize different sizes of QDs^38^, However, due to the insignificant limitations of synthesizing condition, the exact size of QDs can’t be attained; thus the energy levels of QDs is broadened inhomogeneously as a result of the size distribution of QDs^20^ during the solution process method. Generally, as it is seen in Fig. 2(A), the IHB of energy levels has to be considered which is modeled by Gaussian function^20,23,53^. The FWHM of the Gaussian function can take specific values as Γ_G_. In addition, the effect of carrier-carrier and phonon-carrier scattering leads to the homogeneous broadening (HB) of energy levels which is presented by Lorentz shape^20^.

One can realize from Fig. 2(B) that E-R relates to energy versus QD size; also, the suitable radius of QDs shows the resonant energies corresponding to desired emission wavelength; (0.8 eV and 0.946 relate to 1.55  μm and 1.31  μm, respectively). The diagram of Fig. 2(B) is obtained as the consequence of numerically solving the Schrödinger equation for switchable two-color InGaAs/GaAs QD-laser. As it is shown in Fig. 2(B), proportionate to introduced Γ_G_, there is size distribution over both specific R_1_ and R_2,_ each of which is denoted by ∆R_1_ and ∆R_2_, respectively; note that ∆R_1_ is broader than ∆R_2_. Following this result, achieving an efficient device is possible by the solution process method which provides high accuracy experimental conditions to synthesis a small radius of QD with 0.5 nm deviation and the big one with 0.1nm^40^. As reported, the performance of QD-laser is based on rate equations^20,24,40^, so the analyzing proposed structure in this research by consideration of both IHB and HB of QD’s energy levels is carried out based on developed rate equations. Selective low coverage of QDs in the structure causes the QDs not to have correlation and become isolated from each other^40^.

## Modeling Switchable Two-Color QD-Laser in Rate Equation Framework

According to the last reports, solving the rate equations is the most popular way to analyze the QD-laser dynamics^20,24^. In this model, the rate equations are developed to calculate the characteristics of a two-color QD-laser, including SECs. Needless to say, the QDs without any correlation are isolated from each other and it is supposed that both the electron and hole are captured in pairs at GS of the QDs, which form an exciton; in addition, the escape of carriers from SCH layer is neglected, and the following set of developed rate equation is used just for electrons. Here, we assume two ensembles of QDs, each of which contains n = 1, 2, …, 2 M + 1 groups corresponding to the desired wavelengths. The energy width is described as the mode separation of each group and is given by

1 $$\Delta E=\frac{ch}{2{n}_{r}{L}_{cav}}$$

where *c* and *h* stand for the speed of light in vacuum and Planck’s constant respectively, and ${L}_{cav}$is the length of the cavity. Due to the resonance of all modes, ${L}_{cav}$ is selected relevant to the appropriate case. Therefore, ${L}_{cav}$ equals to the multiple of the cavity length of each group^40^,

2 $${L}_{cav}={L}_{cav1}{L}_{cav2}=\frac{{a}_{1}{\lambda }_{1}}{2{n}_{r}}\frac{{a}_{2}{\lambda }_{2}}{2{n}_{r}}=\frac{a{\lambda }_{1}{\lambda }_{2}}{{(2{n}_{r})}^{2}}$$

where ${L}_{cav1}$ and ${L}_{cav2}$ correspond to the cavity length of first and second groups of QDs’ ensemble. The cavity length should be selected in a way that the coefficients *a*, *a*_*1*,_ and *a*_2_ become integer. It is good to be mentioned that in this equation, only the value of each wavelength is considered and the calculated ${L}_{cav}$ is expressed in  μm^40^. Considering solution process technology, the size of each QD group relates to the emission wavelength of each channel and can be deviated from the value R_1_ or R_2_, leading to the broadening of energy levels in each QD ensembles. The IHB of energy levels can be modeled by the Gaussian function^20^ around the central interband transition energy of each channel, ${E}_{1}$ and ${E}_{2}$, which is defined as

3 $$\begin{array}{c}{G}_{i}({E}_{i,n})=\frac{1}{\sqrt{2\pi {\xi }_{0}}}\exp \left(\frac{-{({E}_{i,n}-{E}_{i})}^{2}}{2{\xi }_{0}^{2}}\right)\\ \,i=1,2\,\& \,n=1,2,\ldots ,2M+1\end{array}$$

Also, the FWHM of IHB equals to ${\Gamma }_{G}=2.35{\xi }_{0}35$. The energy ${E}_{n,i}$ is given by,

4 $${E}_{i,n}={E}_{i}-(M-n)\Delta E\,i=1,2\,\& \,n=1,2,\ldots ,2M+1$$

The main mode of each group is defined as ${E}_{1}={E}_{1,M}$ and ${E}_{2}={E}_{2,M}$ that relate to 1.55 μm and 1.31 μm, respectively. In addition, the effect of temperature and various scattering mechanisms including carrier-carrier and phonon-carrier scattering leads to HB, which has a Lorentzian lineshape function^20^ as defined by

5 $${ {\mathcal B} }_{i,mn}({E}_{i,m}-{E}_{i,n})=\frac{\hslash {\varGamma }_{B}/\pi }{{({E}_{i,m}-{E}_{i,n})}^{2}+{(\hslash {\varGamma }_{B})}^{2}}\,i=1,2$$

According to *B*_*i,mn*_ the FWHM is 2*ℏΓ*_*B*_ in which *Γ*_*B*_ is the polarization dephasing or scattering rate^20^. In this equation ${E}_{1,m}$, ${E}_{1,n}$ *(*${E}_{2,m}$, ${E}_{2,n}$*)* refer to the energy of *m*th mode and *n*th group of big QDs, relevant to channel1 (small QDs corresponding to channel2). The QD laser’s linear optical gain is based on density matrix theory and it is represented by

6 $${g}_{i,mn}=\frac{2\pi {q}^{2}\hslash {N}_{Di}}{c{n}_{r}{\varepsilon }_{0}{m}_{0}^{2}}\frac{{|{P}_{i}|}^{2}}{{E}_{i,n}}(2{P}_{GSi,n}-1){G}_{i({E}_{i,n})}{ {\mathcal B} }_{i,mn}({E}_{i,m}-{E}_{i,n})\,i=1,2$$

where *n*_*r*_ is the refractive index and ${N}_{D1}\,({N}_{D2})$ is the density of QDs in channel1 (channel2) which is given by ${\xi }_{i}={N}_{Di}{V}_{Di}$; here *i* = 1, 2 and ${\xi }_{1}\,({\xi }_{2})$ is QD’s coverage of channel1 (channel2), and the volume of a QD with a radius of *R*_1_ (*R*_2_) is ${V}_{D1}\,({V}_{D2})$. The sizes of *R*_1_ = 3.85 nm and *R*_2_ = 2.47 nm determine the lasing wavelength at 1.55 μm and 1.31 μm, respectively. When the HB of energy levels is small in comparison with IHB, the different sizes of QD groups have been spatially isolated from each, leading the optical gain of lasing modes independent of other modes. In better words, when the HB is approximately comparable to IHB, the resonant and non-resonant modes whose energy levels lie within the amount of HB have been considered in the optical gain of lasing modes. The occupation probability in the GS and ES of the QD’s ensembles is in accordance with Pauli’s exclusion principle which is defined by

7 $${P}_{GSi,n}=\frac{{N}_{GSi,n}}{2{N}_{Di}{V}_{A}{G}_{i}({E}_{i,n})}\,i=1,2$$

8 $${P}_{ES1,n}=\frac{{N}_{ES1,n}}{2{N}_{D1}{V}_{A}{G}_{1}({E}_{1,n})}$$

where *N*_*GS1*_ and *N*_*GS*2_ are the carrier numbers of GS for channel1’s QDs and channel2’s QDs, respectively; also *N*_*ES*1_ is the carrier number of ES of channel1’s QDs. The active region’s volume is *V*_*A*_, and the QDs’ densities of each channel are given by ${N}_{D1},{N}_{D2}$. In the optical gain formula, ${|{P}_{i}|}^{2}$ is the transition moment matrix element which is defined as

9 $${|{P}_{i}|}^{2}={|{I}_{cv}|}^{2}{M}_{b}^{2}$$

According to the above formula, ${I}_{cv}$ defines the overlap integral between the envelope functions of both a hole and an electron for each channel’s QDs. *M*_*b*_ is got from *k.p* theory with the first-order interaction between the conduction and valence bands. The transition matrix element is given as follows, in which *E*_*g*_ is the bandgap, Δ is the spin-orbit, and ${m}_{e}^{\ast }$ is the electron effective mass^20^.

10 $${M}_{b}=\frac{{m}_{0}^{2}}{12{m}_{e}^{\ast }}\frac{{E}_{g}({E}_{g}+\Delta )}{{E}_{g}+\frac{2\Delta }{3}}$$

According to defined relations, as stated above, the rate equations of switchable two-color QD-laser by SEC are expressed by

11 $$\frac{d{N}_{si}}{dt}=\frac{{I}_{i}}{q}-\frac{{N}_{si}}{{\tau }_{si}}+\frac{{N}_{sci}}{{\tau }_{sc,e}}-\frac{{N}_{si}}{{\tau }_{sr}}\,i=1,2$$

12 $$\frac{d{N}_{sci}}{dt}=\frac{{N}_{si}}{{\tau }_{si}}-\frac{{N}_{sci}}{{\tau }_{sc,e}}-\frac{{N}_{sci}}{{\tau }_{scr}}-\frac{{N}_{sci}}{{\bar{\tau }}_{tun,i}}\,i=1,2$$

13 $$\frac{d{N}_{GS1,n}}{dt}=\frac{{N}_{sc1}}{{\tau }_{tn,1}}{G}_{1}({E}_{1,n})-\frac{{N}_{GS1,n}}{{\tau }_{Gr}}-\frac{{N}_{GS1,n}}{{\tau }_{G\to E}}(1-{P}_{ES1,n})+\frac{{N}_{ES1,n}}{{\tau }_{E\to G}}(1-{P}_{GS1,n})-\frac{c\Gamma }{{n}_{r}}\mathop{\sum }\limits_{m=1}^{2M+1}{g}_{1,mn}{S}_{1,m}$$

14 $$\frac{d{N}_{ES1,n}}{dt}=\frac{{N}_{GS1,n}}{{\tau }_{G\to E}}(1-{P}_{ES1,n})-\frac{{N}_{ES1,n}}{{\tau }_{E\to G}}(1-{P}_{GS1,n})-\frac{{N}_{ES1,n}}{{\tau }_{Er}}$$

15 $$\frac{d{N}_{GS2,n}}{dt}=\frac{{N}_{sc2}}{{\tau }_{tn,2}}{G}_{2}({E}_{2,n})-\frac{{N}_{GS2,n}}{{\tau }_{Gr}}-\frac{c\Gamma }{{n}_{r}}\mathop{\sum }\limits_{m=1}^{2M+1}{g}_{2,mn}{S}_{2,m}$$

16 $$\frac{d{S}_{i,m}}{dt}=\frac{\beta {N}_{GSi,n}}{{\tau }_{Gr}}+\frac{c\Gamma }{{n}_{r}}\mathop{\sum }\limits_{n=1}^{2M+1}{g}_{i,mn}{S}_{i,m}-\frac{{S}_{i,m}}{{\tau }_{p}}\,i=1,2$$

Note that, *I*_*1*_ and *I*_2_ are injected currents into SECs in channel1 and channel2, respectively. Here, *q* is the electron charge, ${N}_{s1}({N}_{s2})$ is the carrier number of SCH layer of channel1 (channel2), ${N}_{sc1}\,({N}_{sc2})$ is the carrier number of SEC related to channel1 (channel2), and ${S}_{1,m}\,({S}_{2,m})$ are the photon number of *m*th mode of QDs corresponding to channel1 (channel2). The spontaneous emission coupling efficiency is defined by β. The lifetime of injected carriers from the SCH layer into SEC is defined by *τ*_*si*_, and the *τ*_*tun1*_; the tunneling time of injected carrier from SEC into relevant GS level of QDs is *τ*_*tun2*_ and it will be explained in the next section. Diffused carriers are recombined in the SCH layer with *τ*_*sc,r*_. The carrier recombination in the SEC is given by *τ*_*sr*_. The *τ*_*Gr*_, *τ*_*Er*_ are non-radiative recombination of carriers in the QDs energy levels. The carrier escape rate from the SCH layer is *τ*_*sc,e*_. The channel1’s QDs contain two states (i.e. GS and ES); so the carrier relaxation rate from ES to GS and carrier re-excitation from GS to ES in the big QD that are defined by ${\tau }_{E\to G}$ and ${\tau }_{G\to E}$ as follow^23^

17 $${\tau }_{E\to G}^{-1}=(1-{P}_{GS1,n}){\tau }_{E\to G,0}^{-1}$$

18 $${\tau }_{G\to E}^{-1}=(1-{P}_{ES1,n}){\tau }_{G\to E,0}^{-1}$$

Here, ${\tau }_{E\to G,0}^{-1}$ and ${\tau }_{G\to E,0}^{-1}$ are the initial relaxation and re-excitation rates when the GS and ES are empty, respectively. The initial relaxation and re-excitation time are related by^23^

19 $${\tau }_{G\to E,0}={\tau }_{E\to G,0}{e}^{\frac{q({E}_{ES}-{E}_{1})}{KT}}$$

where (${E}_{ES}-{E}_{1}$) is the energy difference between GS and ES in big QD. The photon emission of each channel is caused by the radiative recombination of carriers in both big and small QDs, and the photon lifetime of the cavity is defined by *τ*_*p*_ as follow^20^

20 $${\tau }_{p}^{-1}=\frac{c}{{n}_{r}}\left({\alpha }_{i}+\frac{1}{2{L}_{cav}}\,\mathrm{ln}\,\frac{1}{{r}_{1}{r}_{2}}\right)$$

where *r*_1_, *r*_2_ are the cavity mirrors reflectivity and *α*_*i*_ is the internal loss. As mentioned above, the advantage of the proposed structure of this paper is the possibility of lasing at two emission wavelengths from both channels simultaneously or individually; therefore, the output power can be calculated from independent channels. Note that the evaluated output power of the laser is given from one of the mirrors as follow

21 $${P}_{i,m}=\frac{c{E}_{i,m}{S}_{i,m}}{2{n}_{r}{L}_{cav}}\,\mathrm{ln}\,\frac{1}{r}\,i=1,2$$

here the emitted photon energy from channel1 is *E*_*1,m*_ with the main energy of 0.8 eV; also, *E*_*2,m*_ equals to 0.946 eV as the main energy of channel2. The mirror reflectivity is *r*, which can be substituted with *r*_1_ or *r*_2_.

## Selective Energy Contacts

Achieving to switchable two-color QD-laser is possible by using SECs, which can inject the electrons into the certain QDs. In addition, coupling the QW leads to the improvement of the fast transition of the carrier^44^. In this model, utilizing two different sizes of QDs in the active region of QD-laser results in two-channel emission wavelengths while the lasing from each channel can be controlled by the independent SECs as an injection of carriers. Effective injections of carriers collection from SECs to QDs provide enhancement performance of the structure.

The transition of the carriers through QW to QDs is defined by tunneling rate, which is controlled by the material parameters and thickness of the barriers^45^. Also, the thin enough barrier layers lead to effective tunneling through the SECs to QDs. Here, the QW and QDs are selected InGaAs, where the barriers are AlAs. Also, the energy levels of the QWs are proportional to QD’s GS, i.e.; the thickness of the SEC should be selected in a way that the energy levels of SEC and QD’s GS are equal.

**Figure 3:**
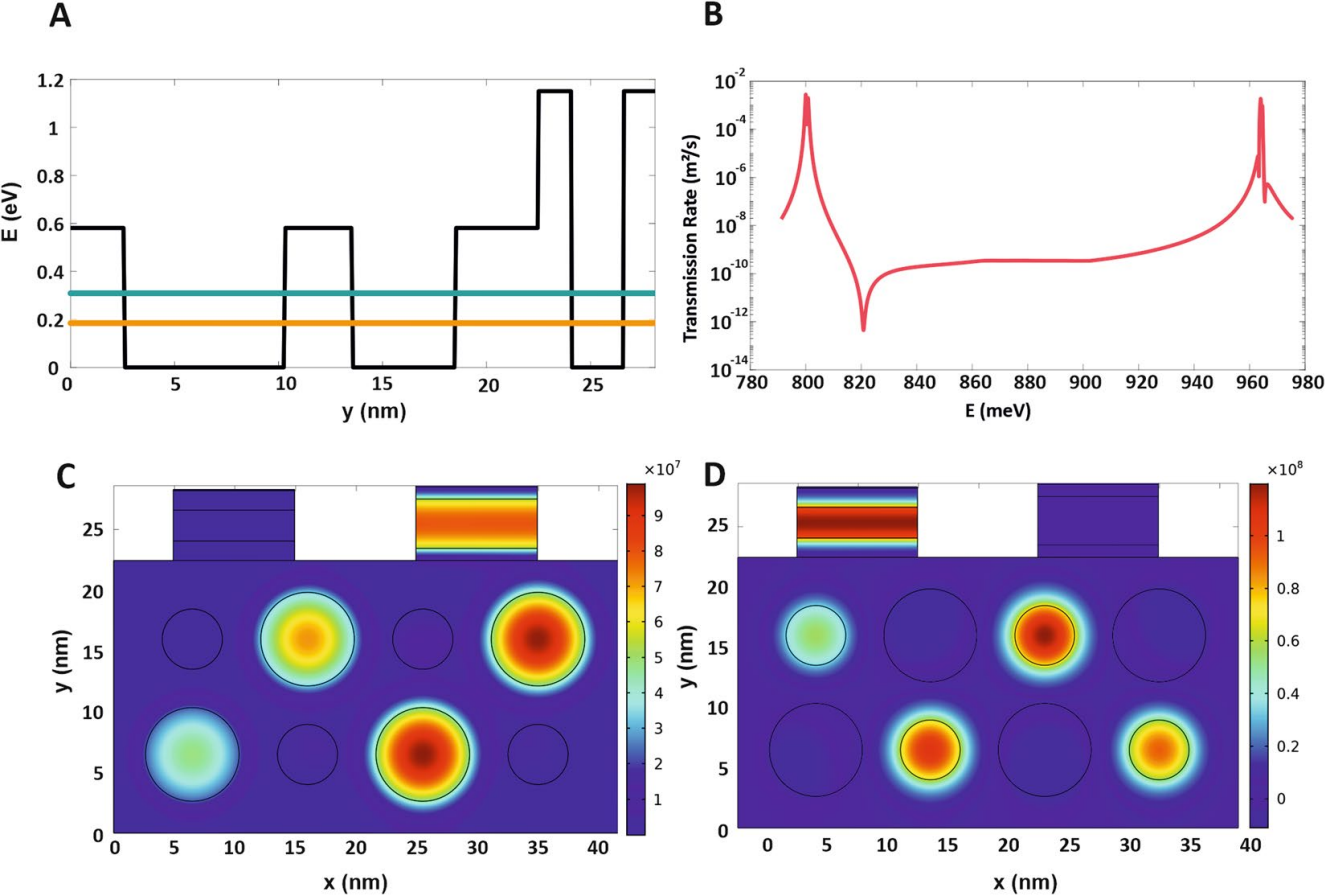
The modal analysis of two-color QD-laser and transmission rate. (**A**) The potential energy along y-direction at constant x-position (black line) and the Eigen-energy of QD-array respect to conduction band edge. The orange and green solid lines correspond to big and small QDs, respectively. (**B**) The transmission rate for carriers injected from SECs tunneled through SECs and QDs. (**C**) The normalized wave-function corresponding to Eigen-energy of big QDs coupled to right SEC and (**D**) small QDs coupled to left SEC. These schematics indicate the electron Eigen-energy of both small and big QD which is summed with hole Eigen-energy and band gap of InGaAs to achieve the resonant energies of 0.8 eV and 0.946 eV corresponding to the emission wavelength 1.55 μm and 1.31 μm, respectively.

The modal analysis of a switchable two-color QD-laser has been done by solving the Schrödinger equation using the Finite Element Method (FEM). The Eigen-energy and corresponding Eigen-function of the system and also the tunneling rate has been calculated. It is good to be mentioned that continuous Periodic Boundary Condition (PBC) is selected for boundaries along the x-axis for active region and SECs. The Eigen-energy of structure respect to the conduction band edge is illustrated in Fig. 3(A). The orange and green solid line corresponds to big and small QDs, which is coupled to relating SECs. The transmission rate of injected electrons from each SEC to QDs along the y-axis has been calculated and depicted in Fig. 3(B). As shown in this figure, there are two peaks corresponding to the main mode of each QD group at 0.8 eV and 0.946 eV. The wavefunction of each QD group coupled to relevant SEC has been shown in Fig. 3(C),(D) for channel1 and channel2, respectively. According to Fig. 3(C),(D) the electrons injected from the right (left) SEC coupled to big (small) QDs and tunneled between QDs. The tunneling time ${\tau }_{tn,i}$ is defined as follows, which ${T}_{trans}$ is the transmission rate of carriers and *N*_*SDi*_ is the surface density of QDs ensemble^44^.

22 $${\tau }_{tn,i}^{-1}={\tau }_{t0,i}^{-1}(1-{P}_{GS1,n})=\frac{1}{{T}_{trans}{N}_{SDi}}(1-{P}_{GS1,n})\,i=1,2$$

where ${\tau }_{t0,i}^{-1}$ is initial tunneling time when GS is empty. The average carrier tunneling lifetime is ${\bar{\tau }}_{tun,i}^{-1}$ and it is given by

23 $${\bar{\tau }}_{tun,i}^{-1}=\mathop{\sum }\limits_{n=1}^{2M+1}{\tau }_{tn,i}^{-1}{G}_{i}({E}_{i,n})=\mathop{\sum }\limits_{n=1}^{2M+1}{\tau }_{t0,i}^{-1}(1-{P}_{GS1,n}){G}_{i}({E}_{i,n})\,i=1,2$$

## The Dynamic Behavior of Switchable Two-Color QD-Laser

The dynamic behavior of the two-color QD-laser has been simulated by the calculation of the small-signal modulation response^26,51,57^. To this end, a current step has been applied to each SECs as follows

24 $${i}_{i}(t)={I}_{i}U(t)+\Delta {I}_{i}U(t-{t}_{0})\,i=1,2$$

where $U(t)$ is a unit step function, ${I}_{1}$ and ${I}_{2}$ are the bias current of channel1 and channel2, respectively. The bias currents have to be larger than the threshold current of the related channel. The $\Delta {I}_{i}$ ($\Delta {I}_{1}$ and $\Delta {I}_{2}$) is the small step perturbation which is much smaller than threshold current, applied to each channel. The duration of the current step has to be long enough to ensure that the transient response settles down. The improved rate Eqs. (11–16), taking into account the modulated current for each channel mentioned in Eq. (24), have to be solved based on the fourth-order Runge-Kutta method. The small-signal modulation response of a two-color QD-laser can be obtained by the Fourier transform of photon densities considering the small step current.

## The Switchable two-Color QD-Laser utilizing SECs

The switchable two-color QD-laser using SECs has been characterized by solving the improved rate Eqs. (11–16) based on fourth-order Runge-Kutta method, in order to time evaluation of carriers and photon densities. The output power emitted in accordance with the biasing of each channel can be obtained through photon densities at a steady-state. Hence, the lasing spectra relevant to each channel of the proposed QD-laser have been calculated on the steady-state output power of each main mode. Moreover, calculating the steady-state carrier densities in GS of big and small QDs leads to obtaining the gain spectra relating to each channel.

In the suggested model, the re-excitation of carriers are ignored; we assumed at first all the carriers are injected in the SCH layer, then those tunnel from the contact to the QDs; in this assumption, the escape of the carriers from SCH layer is neglected. The utilization of QDs in different sizes, observed in this type of QD-laser, is provided by solution process technology, which is a simple and low-cost method. In this process, the simple condition of the chemical system permits to achieve tunable sizes of QDs that are used in the high-performance devices. In the solution process, the QD size deviation of around 0.1 nm can be possible in the appropriate experimental condition of the chemical procedure, which causes the broadening of origin energy. Therefore, the size fluctuation of QDs always occurs in the QDs ensemble, and efficient tunneling will be done within a certain range of QDs’ size. For this reason, the IHB is assumed about 5 meV. In addition, the HB is assumed 20 meV at room temperature. The parameters of modeled tunneling-injection QD-laser are given in Table 1.

**Table 1:** Simulation parameters for the proposed model.

Symbol	Value	Description
${R}_{1}$	3.85(nm)	Quantum dot radius
${R}_{2}$	2.47(nm)	Quantum dot radius
${V}_{Di}\,({m}^{3})$	$\frac{4}{3}\pi {R}_{i}^{3}$	Volume of a QD
$\varGamma$	6%^20^	Optical confinement factor
${r}_{1},\,{r}_{2}$	0.3, 0.9^20^	Mirrors reflectivity
${\alpha }_{i}$	600(cm^−1^)^20^	Internal loss
$d$	10( μm)^20^	Stripe width
${V}_{A}$	$1.62\times {10}^{-16}$(${m}^{3}$)	The volume of the active region
${L}_{cav}$	812.2( μm)	Cavity length
$\beta$	10^−4 ^^20^	Spontaneous emission coupling efficiency
${n}_{r}$	3.5^20^	Refractive index
${E}_{g}$	0.587(eV)	The bulk semiconductor’s bandgap of QD
${m}_{e}^{\ast }$	$0.034{m}_{0}$	The electron effective mass of QD
${E}_{g\_QW}$	2.16(eV)^58^	The bulk semiconductor’s bandgap of QW
${m}_{e\_QW}^{\ast }$	$0.022{m}_{0}$^58^	The electron effective mass of QW
${\tau }_{si}$	1(ps)^20^	diffusion of the carriers in the SCH layer
${\tau }_{sr}$	2.8(ns)^20^	Carrier recombination time in the SCH layer
${\tau }_{sc,r}$	2.8(ns)^20^	Carrier recombination time in the SECs
${\tau }_{E\to G}$	3(ps)	Initial carrier relaxation lifetime
${\tau }_{Gr}$ ${\tau }_{Er}$	2.8(ns)^20^	Carrier recombination lifetime in the QD

In this paper, the switchable two-color QD-laser using SECs is investigated by assuming the two wavelengths, 1.31 μm and 1.55 μm, which have special positions in telecommunication applications. The radiuses of spherical QDs in the active region are 3.85 nm and 2.47 nm, and the wavelength difference of about 240 nm is an appropriate magnitude to the independent lasing for these wavelengths without overlapping. The prominent idea in this work is introducing the switchable QD-laser, which can provide emission lasing just at one wavelength or at two wavelengths simultaneously. To prove this idea in the following, the output characteristics of this QD-laser is investigated.

Based on described rate equations in the previous section, the output power intensity depends on QD coverage and the photon numbers, which can be controlled by injection current at each wavelength. Thus, the high intensity of output power for two different wavelengths is attained at specific QD coverage, so the coverage of big QDs is considered ξ_1_ = 0.07, whereas the small one is ξ_2_ = 0.05. Furthermore, at 1.31  μm, the output power value gets efficient due to the effective carrier transition of channel2 (1.31 μm) in comparison with channel1 (1.55 μm) at I_th_ = 14 mA. Also, for both channels, the FWHM of HB and IHB are assumed to 2*ℏΓ*_*B*_ = 20 meV and Γ_G_ = 5 meV, respectively.

**Figure 4:**
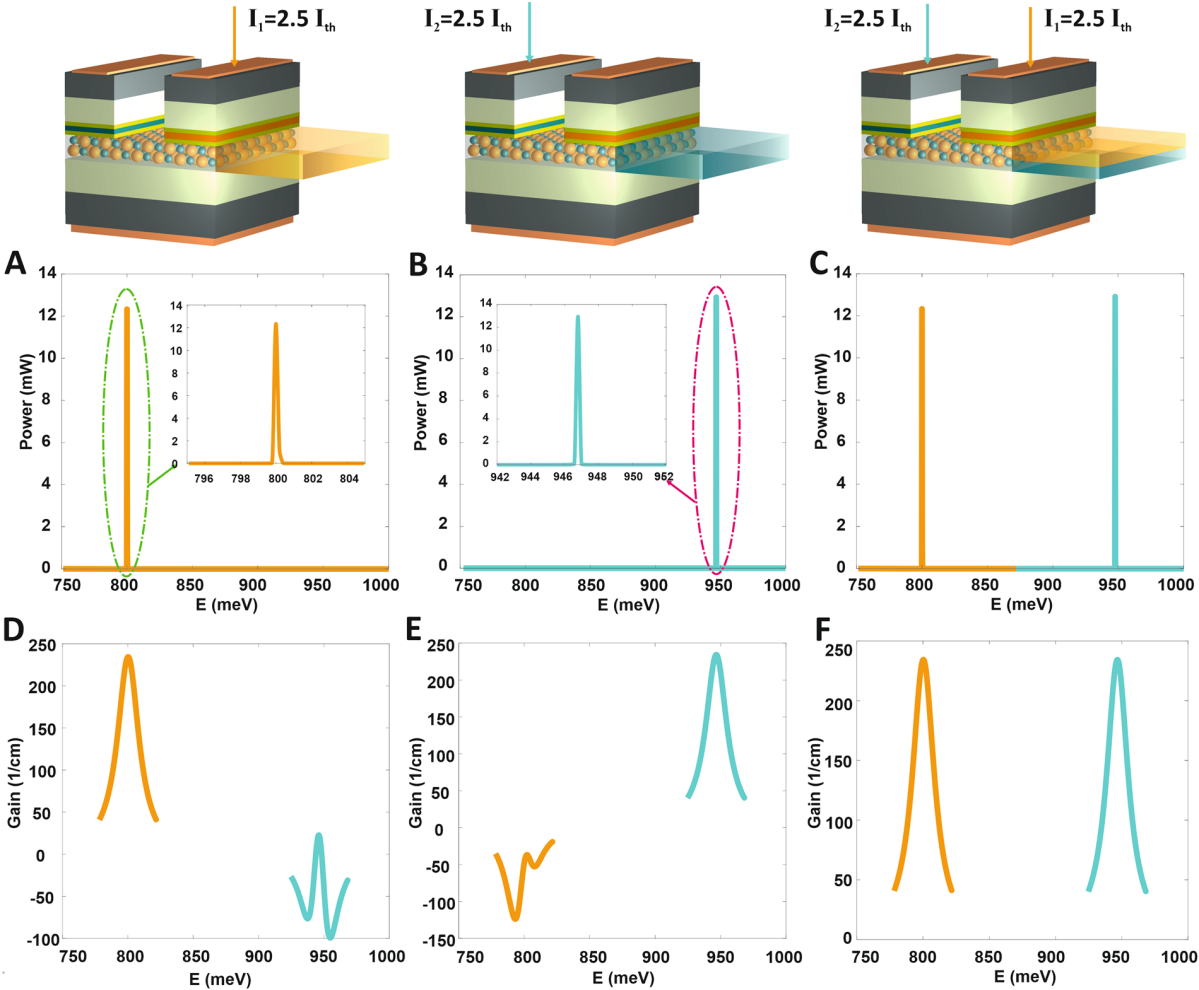
The light emission spectrum and optical gain spectrum of switchable two-color QD-laser. (**A**) The output power spectrum for big QDs relates to 1.55  μm when the current only is injected to channel1 and (**B**) for small QDs relates to 1.31  μm when the current only is injected to channel2. (**C**) The orange and green solid lines correspond to big and small QDs output powers spectrum, respectively. The injection current is carried out simultaneously in both channels. (**D**) The orange solid curve is the optical gain spectrum of big QDs and relates to 1.55  μm, as the green curve relates to small QDs with no injection current. (**E**) The small QDs optical gain spectrum is shown by green curve and relates to 1.31  μm, and the orange one relates to big QDs with no injection current. (**F**) The orange and green solid lines correspond to big and small QDs optical gain spectrum, respectively. The injection current is carried out simultaneously in both channels. The injected current only in one channel (**A**) and (**B**) or in both of them (**C**) equal to I_1_ = 2.5I_th,_ I_2_ = 2.5I_th_. Note that in both channel I_th_ = 14 mA. The QD coverage of big and small QDs are ξ_1_ = 0.07 and ξ_2_ = 0.05. In both channel1 and channel2 the FWHM of HB and IHB are set to 2*ℏΓ*_*B*_ = 20 meV and Γ_G_ = 5 meV, respectively. The resonant energies of 0.8 eV and 0.946 eV correspond to the emission wavelength 1.55 μm and 1.31 μm, respectively.

Figure 4(A) illustrates the output power intensity of big QDs ensemble when the current is injected just to the related contact; in other words, there is no current injection to the other contact, so the lasing only occurs at 1.55 μm. The same as the previous performance which was applicable for its contact and is shown in Fig. 4(B), lasing occurs just at 1.31 μm. The super-strength of this suggested model is the selectivity of emission wavelength, in which the existence of different sizes of QDs and the related QW contacts allow controlling the lasing emission wavelength. Figure 4(C) shows the output power when the current is injected into both contacts and the lasing occurs in two wavelengths simultaneously so we can see the output power intensity in both of them, the intensity of output power in channel1 and channel2 equals to 12.3 mW and 12.95 mW. The Fig. 4(D),(E) shows the optical gain spectrum of big QDs (small QDs) when the injection current to channel1 (channel2) is I_1_ = 2.5I_th_ = 35 mA. Since there is no current injection into channel2 (channel1), so the negative gain can be noticed which is determined by the green curve (orange curve). Finally, Fig. 4(F) depicts the optical gain spectrum at both 1.55  μm and 1.31  μm when the current is injected in both channels simultaneously.

**Figure 5:**
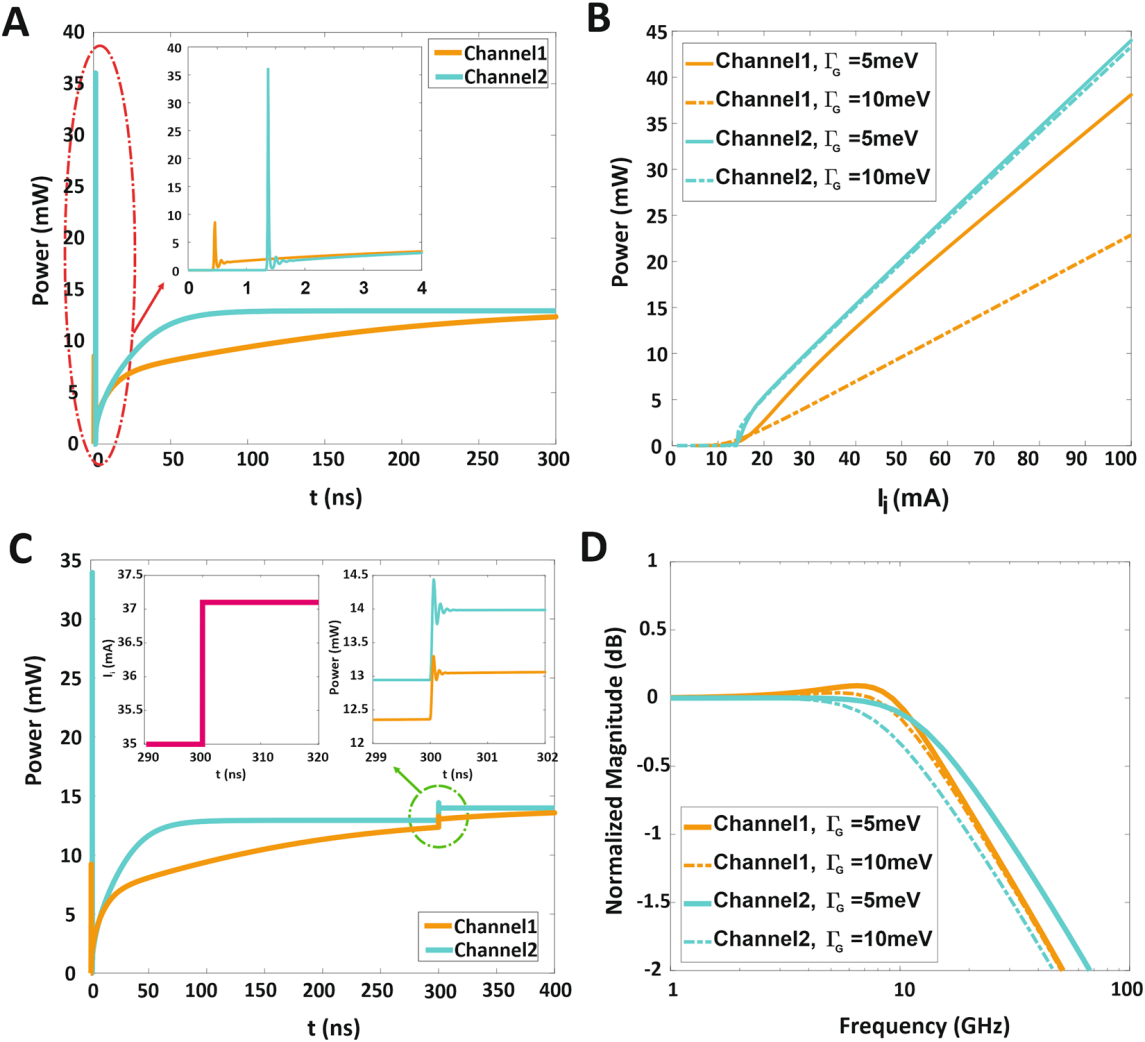
The transient response and output power of two-color QD-laser. (**A**) The transient response of output power for 1.55  μm and 1.31  μm at ξ_1_ = 0.07, ξ_2_ = 0.05 and the FWHM of IHB are set to *Γ*_G_ = 5 meV, respectively. (**B**) The output power versus injected current for the central lasing mode at two wavelengths 1.55  μm and 1.31  μm, I_i_ sets to I_1_ and I_2_, also the FWHM of IHB is considered Γ_G_ = 5 meV and 10 meV. (**C**) The transient response of output power results for applying the step perturbation, ∆I_i_ = 0.05 I_b_, the bias current is assumed 2.5I_th_ and I_th_ = 14 mA. (**D**) Modulation response for 1.55  μm and 1.31  μm with IHB as a parameter (Γ_G_ = 5 meV and 10 meV). In All figures (**A–D**) the FWHM of HB is 2*ℏΓ*_*B*_ = 20 meV and ξ_1_ = 0.07, ξ_2_ = 0.05; also the orange and green lines correspond to big and small QDs, respectively.

In Fig. 5(A) by assuming ξ_1_ = 0.07 and ξ_2_ = 0.05, at injected current I_1_ = I_2_ = 2.5I_th_ (I_th_ = 14 mA) the transient response of output power characteristics for the switchable two-color QD-laser using SECs are illustrated for both big QDs (orange line) and small QDs (green line) corresponding to the wavelengths of 1.55 μm and 1.31 μm, respectively while the FWHM of HB and IHB are considered to 2*ℏΓ*_*B*_ = 20 meV and Γ_G_ = 5 meV. As shown in this figure, the turn-on delay time of big QDs is smaller than the small ones.

The output power as a function of injected currents for switchable two-color QD-laser using SECs has been shown in Fig. 5(B). Here, I_i_ sets to I_1_ and I_2_ which relates to channel1 (1.55  μm) and channel2 (1.31  μm). The orange solid curve relates to 1.55  μm and the green solid one to 1.31  μm. By increasing the injected current, the output power corresponding to each wavelength is enhanced linearly with different slope efficiency. Here, as it is seen, the threshold current of both channels is almost equal and it is about 14 mA, despite the different considered coverage of big and small QDs, where ξ_1_ = 0.07 and ξ_2_ = 0.05. In other words, applying injection current in both channels simultaneously where their QD coverage is different, leads to the population inversion occurs at the same current in both big and small QD group. As another impact, the output power variation with injection current has been investigated for two different FWHM of IHB (Γ_G_ = 5 meV and 10 meV). The comparison of solid and dashed orange curves (channel1) in Fig. 5(B) demonstrates that the output power is enhanced linearly by increasing the injected current in both cases but their slope efficiency is different. Hence, at special injected current, while Γ_G_ = 10 meV the output power value is smaller than it in Γ_G_ = 5 meV. When the FWHM of IHB is considered 10 meV, the more QD group should have lied within the HB of the central group (corresponds to the resonant mode of each QD group), but considering narrow transmission rate cannot satisfy all the QDs, and the output power decreases. Furthermore, the existence of both ES and GS in big QD leads to carrier transition between them, and due to the lack of carriers in GS, the output power can be decreased. Conversely, in channel2, the output power intensity for both Γ_G_ = 5 meV and 10 meV gets the same slope efficiency. It is presumed that the small QD has just GS; thus the carriers emitting into the central mode become prominent; this leads to the stability of output power while increasing FWHM of IHB from 5 meV to 10 meV.

Additionally, Fig. 5(C) illustrates the transient response of channel1 (1.55  μm) and channel2 (1.31  μm). In order to calculate, the step perturbation (∆I_i_ = 0.05I_b_ and I_b_ = 2.5I_th_) is applied in both channels after the output power reaches steady-state at t = 300 ns; note that it is much smaller than threshold current (the pink curve inset of Fig. 5(C)). Also, in both channels, variation of output power, in comparison with its initial value, should be restricted to very small values. Evidently, taking into account the long enough length of the current step leads to transient response settles down (solid orange and green corresponding to 1.55  μm and 1.31  μm, respectively). In continuation to the explanation, Fig. 5(D) shows the numerical results of the modulation responses in both channels which are calculated analytically from Eq. (24), by assuming Γ_G_ = 5 meV and 10 meV, while the bias current is considered 2.5I_th_ for both channels (1.55  μm and 1.31  μm); also ξ_1_ = 0.07 and ξ_2_ = 0.05. Here in figure, solid and dashed orange curves correspond to Γ_G_ = 5 meV and 10 meV of channel1 (1.55  μm), respectively.

As it is observed in this figure, the QD size distributions (IHB) leads to small variation over the modulation bandwidth which can be ignored (the modulation bandwidths equal about 11 GHz). It should be mentioned that in channel1 if FWHM of IHB takes a high value, the output power declines. So, achieving the efficient operation for channel1 is possible by choosing the suitable FWHM of IHB which can be controlled by ∆R_1_. Consequently, in channel1, the size of QD may have a deviation of ∆R_1_ = 0.12 nm for QD radii of R_1_ = 3.85 nm which causes the broadening of origin energy. In this case, Γ_G_ = 5 meV and it is worthy of mention that, by using the solution process method, it is possible to obtain very uniform and precise QDs with 0.12 nm size deviation.

Besides, in Fig. 5(D), the solid and dashed green curves are depicted the modulation response behavior of channel2 (1.31  μm). Although increasing the QD size distributions (IHB) can vary the modulation bandwidth, the output power still remains stable. Therefore, the comparison of curves leads to the observation of 11 GHz modulation bandwidth at Γ_G_ = 5 meV while 11 GHz belongs to Γ_G_ = 10 meV. Considering deviation about ∆R_2_ = 0.1 nm for a specific small QD radius R_2_ = 2.47 nm and the possibility of achieving the unique size by solution process technology, leads to select Γ_G_ = 5 meV for channel2.

**Figure 6:**
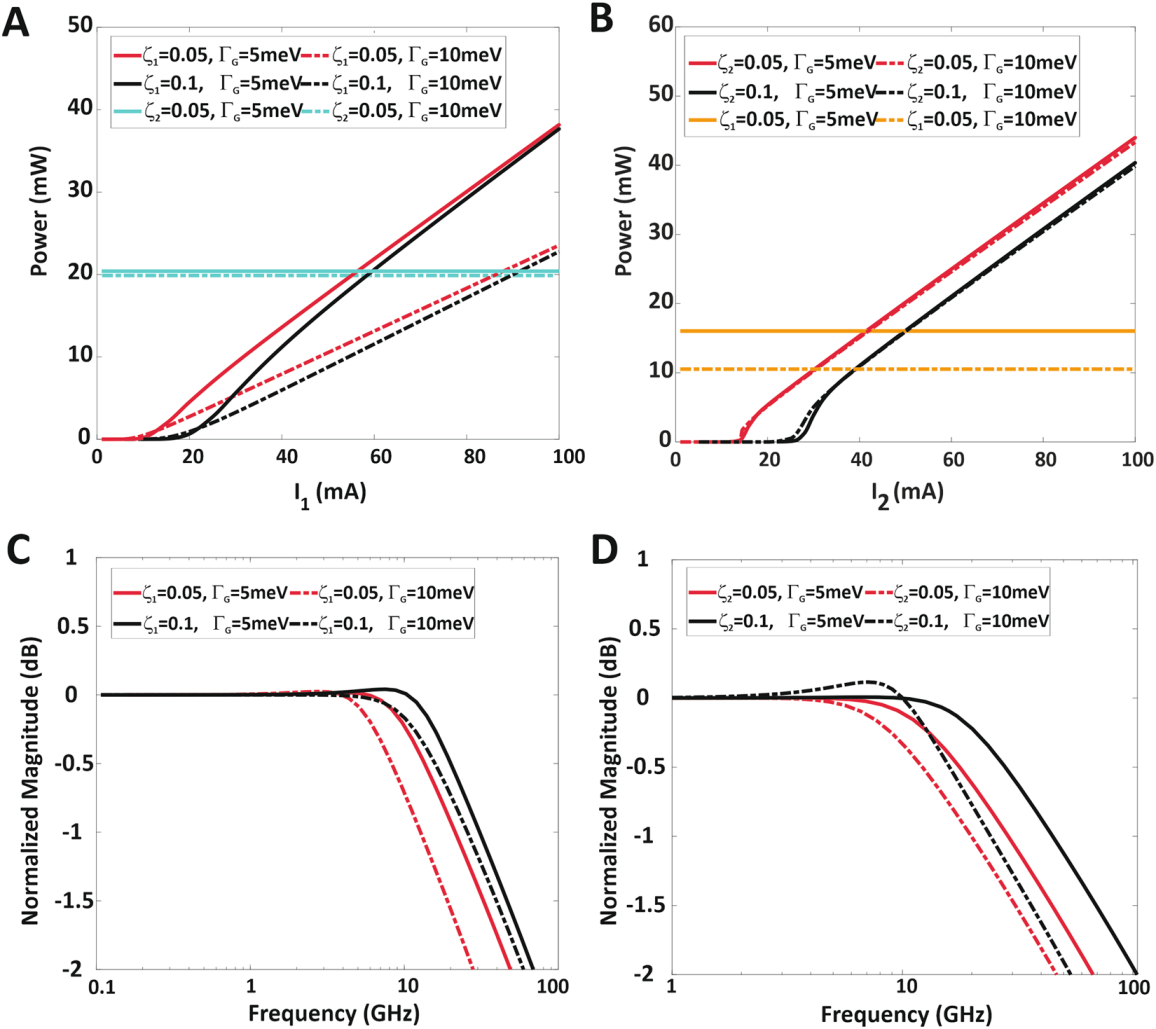
The output power and modulation response of two-color QD-laser for different ξ and Γ_G_ (**A**) The output power versus injected current for the central lasing mode at 1.55  μm radiation wavelength when its QD coverage (big QD) is ξ_1_ = 0.05 and 0.1 at Γ_G_ = 5 meV and 10 meV (**B**) The output power versus injected current for the central lasing mode at 1.31  μm radiation wavelength when its QD coverage (small QD) is ξ_2_ = 0.05 and 0.1 at Γ_G_ = 5 meV and 10 meV (**C**) Modulation response of 1.55  μm (big QD) with Γ_G_ = 5 meV and 10 meV for ξ_1_ = 0.05 and 0.1. (**D**) Modulation response of 1.31  μm (small QD) with Γ_G_ = 5 meV and 10 meV for ξ_2_ = 0.05 and 0.1 .The transient response of output power results in applying the step perturbation, ∆I_i_ = 0.05I_b_, the bias current is assumed 2.5I_th_. In All figures (**A–D**) the FWHM of HB is 2*ℏΓ*_*B*_ = 20 meV.

The output power intensity corresponding to the wavelengths of 1.55  μm and 1.31  μm as a function of injected current for various QD coverage at both Γ_G_ = 5 meV and 10 meV is demonstrated in Fig. 6(A),(B), respectively. Considering various numbers of coverage for big QDs (channel1 corresponds to 1.55  μm) and small QDs (channel2 corresponds to 1.31  μm) at constant injected current, due to the lack of carriers, the number of occupied big (small) QDs, lying within the scope of central mode of big (small) QDs, decreases when the coverage of big (small) QDs increases; therefore, the corresponding output power intensity reduces, and the threshold current increases as illustrated in Fig. 6(A),(B). In other words, by increasing the coverage of big (small) QDs in both channels, higher injection current is needed to achieve the threshold gain and start lasing. As Fig. 6(A),(B) depicts, in all cases by increasing the injection current, the output power increases linearly.

In Fig. 6(A), the current injected in channel1 varies gradually and the current injection to channel2 is constant. Hence, by considering ξ_2_ = 0.05, as it is seen, the solid and dashed green lines for both Γ_G_ = 5 meV and 10 meV, shows the output power has constant value equaling to 20 mW. As explained earlier, by comparing the different slop efficiency of output power curves at Γ_G_ = 5 meV and 10 meV, it is revealed that high value of IHB (Γ_G_ = 10 meV) leads to more QD group lying within the HB of central group, while the narrow transmission rate cannot satisfy all the QDs, so at the same QD coverage, the output power intensity of Γ_G_ = 10 meV is lower than it for Γ_G_ = 5 meV.

Similarly, the comparison of curves in Fig. 6(B) demonstrates that the coverage of big QDs is constant (ξ_1_ = 0.05) and the current injection into channel1 is constant (solid and dashed orange lines show the output powers at Γ_G_ = 5 meV and 10 meV, which equal to 10.5 mW and 16 mW, respectively). As described previously, increasing the coverage of small QDs causes threshold current increase; also, the slope efficiency of curves for both Γ_G_ = 5 meV and 10 meV isn’t different due to the existence of only GS in small QDs and prominent carrier emitting into the central mode.

Based on the transient response result, the modulation response is analyzed for different coverage of big and small QDs for channel1 (1.55  μm) and channel2 (1.31  μm) that are depicted in Fig. 6(C),(D), respectively. As shown in both figures, the modulation bandwidth depends on QD coverage and by their variation, it can change. Therefore, numerical results demonstrate that by increasing QD coverage, a high value of modulation bandwidth can be obtained in both channels. As mentioned above, calculating the modulation behavior is carried out by applying very small step perturbation, which is determined by bias current ((∆I_i_ = 0.05I_b_) and depends on threshold current (I_b_ = 2.5I_th_).

On the other hand, by increasing QD coverage, higher injection current is needed to achieve the threshold gain, so the threshold current increases. In better words, broader bandwidth can be achieved by high bias current as a result of increasing QD coverage, which leads to a high threshold current. In both Fig. 6(A),(B), the FWHM of HB equals to 20 meV; also Γ_G_ = 5 meV and 10 meV. Both figures illustrate the QD size distributions (FWHM of IHB equal to 5 meV and 10 meV) that lead to small variation over the modulation bandwidth. So, the solution process is a low-cost method which can easily control the QD radii and provide unique QDs with minimum size deviation to achieve high-performance device. The resultant maximum value of modulation bandwidth in channel1 (Fig. 6(A)) at ξ_1_ = 0.1 and Γ_G_ = 5 meV is about 14 GHz (solid black curve) and for channel2 (Fig. 6(B)) at ξ_2_ = 0.1 with Γ_G_ = 5 meV is about 18 GHz (solid black curve). Also, it should be borne in mind that both of the output emissions can be modulated independently in this proposed structure.

## Conclusion

In this paper, the switchable two-color QD-laser utilizing SECs by considering the solution process technology has been proposed for the first time. The realization of controlling the lasing channels at different wavelengths is possible by utilizing the QWs as separate selective energy contacts. Furthermore, it was shown that two-color lasing at 1.31  μm and 1.55  μm was achieved by implementing two different radii of InGaAs/GaAs quantum dots in the active layer. Moreover, simultaneously or individually lasing of the proposed switchable two-color QD-laser is modeled based on modified rate equations. The investigation was carried out by considering inhomogeneous broadening as a result of the size distribution of QDs and considering tunnel injection of carriers into the QDs via SECs. Besides, the effect of QD coverage and changing FWHM of IHB on the threshold current, output power intensity and modulation response of each lasing channel were discussed. Controlling the QD coverage and FWHM of inhomogeneous broadening leads to provide customized output power peak value and modulation bandwidth. Finally, yet important, in this work, the design of switchable two-color QD-laser using separate SECs capability choosing the lasing channel was proposed, and the solution process method as the realization of this structure was presented. Furthermore, both channels can be modulated independently, which is a significant feature in high-speed data transmission.

## References

[1] Lüdge, K. & Schöll, E. Temperature dependent two-state lasing in quantum dot lasers. *2011 5th Rio La Plata Work. Laser Dyn. Nonlinear Photonics, LDNP 2011*, 10.1109/LDNP.2011.6162081 (2011).

[2] Maximov MV (2013). The influence of p-doping on two-state lasing in InAs/InGaAs quantum dot lasers. Semicond. Sci. Technol..

[3] Markus A (2003). Impact of Intraband Relaxation on the Performance of a Quantum-Dot Laser. IEEE J. Sel. Top. Quantum Electron..

[4] Wang C, Grillot F, Member S, Even J (2012). Impacts of Wetting Layer and Excited State on the Modulation Response of Quantum-Dot Lasers. IEEE J. Quantum Electron..

[5] Korenev VV, Savelyev AV, Zhukov AE, Omelchenko AV, Maximov MV (2013). Effect of carrier dynamics and temperature on two-state lasing in semiconductor quantum dot lasers. Semiconductors.

[6] Wu L (2017). Few-Layer Tin Sulfide: A Promising Black-Phosphorus- Analogue 2D Material with Exceptionally Large Nonlinear Optical Response, High Stability, and Applications in All-Optical Switching and Wavelength Conversion. Adv. Optical Mater..

[7] Huang W (2018). Black-Phosphorus-Analogue Tin Monosulfide: An Emerging Optoelectronic Two-Dimensional Material for High-Performance Photodetection with Improved Stability under Ambient/Harsh Conditions. J. Mater. Chem. C..

[8] Xing C (2017). 2D Nonlayered Selenium Nanosheets: Facile. Synthesis, Photoluminescence, and Ultrafast Photonics. Adv. Optical Mater..

[9] Fan T (2019). Two-Dimensional Non-Layered Selenium Nanoflakes: Facile Fabrications and Applications for Self-Powered Photo-Detector. Nanotechnology..

[10] Xie Z (2019). Revealing of the ultrafast third-order nonlinear optical response and enabled photonic application in two-dimensional tin sulfide. Photonics Res..

[11] Chen, J. *et al*. Advances in Nanomaterials for Photodynamic Therapy Applications. *Biomaterials*., 10.1016/j.biomaterials.2020.119827 (2020). 10.1016/j.biomaterials.2020.119827 32036302

[12] Xie, Z. *et al*. The Rise of 2D Photothermal Materials beyond Graphene for Clean Water Production. *Adv. Sci*., 10.1002/advs.201902236 (2020). 10.1002/advs.201902236 PMC7055570 32154070

[13] Xie Z (2018). Black Phosphorus analogue Tin Sulfide Nanosheets: Synthesis and Application as Near-Infrared Photothermal Agents and Drug Delivery Platforms for Cancer Therapy. J. Mater. Chem. B..

[14] Liang X (2019). Photothermal cancer immunotherapy by erythrocyte membrane-coated black phosphorus formulation. J. Control. Release.

[15] Xing C (2018). Conceptually Novel Black Phosphorus / Cellulose Hydrogels as Promising Photothermal Agents for Effective Cancer Therapy. Adv. Healthcare Mater..

[16] Xie, Z., Yu, L., Xing, C., Qiu, M. & Hu, J. Solar-inspired Water Purification Based on Emerging Two-dimensional Materials: Status and Challenges. *Solar RRL*., 10.1002/solr.201900400 (2019).

[17] Xie Z (2019). Biocompatible Two-Dimensional Titanium Nanosheets for Multimodal Imaging-Guided Cancer Theranostics. ACS Appl. Mater. Interfaces..

[18] Xie Z (2018). Ultrathin 2D Nonlayered Tellurium Nanosheets: Facile Liquid-Phase Exfoliation, Characterization, and Photoresponse with High Performance and Enhanced Stability. Adv. Funct. Mater..

[19] Xing C (2017). Ultra-Small Bismuth Quantum Dots: Facile Liquid- Phase Exfoliation, Characterization, and Application in High-Performance UV-Vis Photo-detector. ACS Photonics..

[20] Sugawara M, Mukai K, Nakata Y, Ishikawa H, Sakamoto A (2000). Effect of homogeneous broadening of optical gain on lasing spectra in self-assembled In_x_Ga_1-x_As/GaAs quatum dot lasers. Phys. Rev. B..

[21] Lv ZR (2015). Dynamic characteristics of two-state lasing quantum dot lasers under large signal modulation. AIP Adv..

[22] Röhm A, Lingnau B, Lüdge K (2015). Ground-state modulation-enhancement by two-state lasing in quantum-dot laser devices. Appl. Phys. Lett..

[23] Sugawara M (2005). Modeling room-temperature lasing spectra of 1.3- μm self-assembled InAs/GaAs quantum-dot lasers: Homogeneous broadening of optical gain under current injection. J. Appl. Phys..

[24] Ghodsi Nahri D (2012). Simulation of output power and optical gain characteristics of self-assembled quantum-dot lasers: Effects of homogeneous and inhomogeneous broadening, quantum dot coverage and phonon bottleneck. Opt. Laser Technol..

[25] Ghodsi Nahri D (2012). Analysis of dynamic, modulation, and output power properties of self-assembled quantum dot lasers. Laser Phys. Lett..

[26] Laser SQ, Yavari MH, Ahmadi V, Member S (2011). Effects of Carrier Relaxation and Homogeneous Broadening on Dynamic and Modulation Behavior. IEEE J. Sel. Top. Quantum Electron..

[27] Sugawara M (1999). Light emission spectra of columnar-shaped self-assembled InGaAs/GaAs quantum-dot lasers: Effect of homogeneous broadening of the optical gain on lasing characteristics. Appl. Phys. Lett ..

[28] Sugawara M (1997). Effect of phonon bottleneck on quantum-dot laser performance. Appl. Phys. Lett..

[29] Markus A (2003). Simultaneous two-state lasing in quantum-dot lasers. Appl. Phys. Lett..

[30] Amini P (2015). High-Performance Solution Processed Inorganic Quantum-Dot LEDs. IEEE Trans. Nanotechnol..

[31] Amini P, Dolatyari M, Rostami G, Rostami A (2015). High Throughput Quantum Dot Based LEDs. *Energy Effic. Improv*. Smart Grid Components.

[32] Yousefabad HG, Matloub S, Rostami A (2019). Ultra-broadband Optical Gain Engineering in Solution-processed QD-SOA Based on Superimposed Quantum Structure. Sci. Rep..

[33] Sargent ETH, Member S (2008). Solution-Processed Infrared Optoelectronics: Photovoltaics, sensors, and sources,. IEEE J. Sel. Top. Quantum Electron..

[34] Park Y, Lim J, Klimov VI (2020). Optically pumped colloidal-quantum-dot lasing in LED-like devices with an integrated optical cavity. Nat. Commun..

[35] Environ E, Debnath R, Sargent EH (2011). Environmental Science Solution-processed colloidal quantum dot photovoltaics: A perspective. Energy Environ. Sci..

[36] Arquer FPGD, Armin A, Meredith P, Sargent EH (2017). Solution-processed semiconductors for next-generation photodetectors. Nat. Rev. Mater..

[37] Chen, Y. *et al*. Fabrication and optical characterization of a flexible colloidal quantum dot laser. *Proc. IEEE Conf. Nanotechnol*. 958–962, 10.1109/nano.2011.6144324 (2011).

[38] Samuel I (2018). Colloidal nanocrystals: Electrifying quantum dots for lasers. Nat. Mater..

[39] Hoogland, S. *et al*. A 1.53 μm colloidal nanocrystal quantum dot laser. *In Conference on Lasers and Electro-Optics and 2006 Quantum Electronics and Laser Science Conference, CLEO/QELS 2006*, 10.1109/cleo.2006.4627687 (2006).

[40] Amini P, Matloub S, Rostami A (2020). Multi-wavelength solution-processed quantum dot laser. Opt. Commun..

[41] Fathpour S, Mi Z, Bhattacharya P (2005). High-speed quantum dot lasers. J. Phys. D: Appl. Phys..

[42] Mi Z, Bhattacharya P, Fathpour S (2005). High-speed 1 . 3 μm tunnel injection quantum-dot lasers. Appl. Phys. Lett..

[43] Han D, Asryan LV (2010). Output power of a double tunneling-injection quantum dot laser. Nanotechnology..

[44] Asryan LV (2017). Effect of pumping delay on the modulation bandwidth in double tunneling-injection quantum dot lasers. Optics Letters..

[45] Han D, Asryan LV, Member S (2009). Effect of the Wetting Layer on the Output Power of a Double Tunneling-Injection Quantum-Dot Laser. J. Lightwave Technol..

[46] Chuang SL, Holonyak N (2002). Efficient quantum well to quantum dot tunneling: Analytical solutions. Appl. Phys. Lett..

[47] Zarinetchi F, Smith SP, Ezekiel S (1991). Stimulated Brillouin fiber-optic laser gyroscope. Opt. Lett..

[48] Ajiya M, Mahdi MA, Hitam S, Mokhtar M (2009). Seamless tuning range based-on available gain bandwidth in multiwavelength Brillouin fiber laser. Opt. Express..

[49] Veselinov K (2009). Spectral Analysis of 1. 55- µm InAs/InP (113) B Quantum-Dot Lasers Based on a Multipopulation Rate Equations Model. IEEE J. Quantum Electron..

[50] Allen CN (2006). External-cavity quantum-dot laser tunable through 1 . 55 μm. Appl. Phys. Lett..

[51] Quantum G, Lasers D, Model AACR (2009). Carrier Relaxation and Modulation Response of 1.3 μm InAs–GaAs Quantum Dot Lasers. J. Lightwave Technol..

[52] Sugawara M, Ebe H, Hatori N, Ishida M (2004). Theory of optical signal amplification and processing by quantum-dot semiconductor optical amplifiers. Phys. Rev. B..

[53] Sugawara M (2002). Quantum-dot semiconductor optical amplifiers for high-bit-rate signal processing up to 160 Gb s-1and a new scheme of 3R regenerators. Meas. Sci. Technol..

[54] Asryan LV, Luryi S (2001). Tunneling-Injection Quantum-Dot Laser: Ultrahigh Temperature Stability. IEEE J. Quantum Electron..

[55] Liang L, Reiss P, Protiere M (2009). Core/Shell Semiconductor Nanocrystals. small..

[56] Mukai K (2000). 1.3 μm CW Lasing Characteristics of Self-Assembled InGaAs–GaAs Quantum Dots. IEEE J. Quantum Electron..

[57] Yavari, M. H., Member, S., Ahmadi, V. & Member, S. Modulation Characteristics of Self-Assembled InAs-GaAs Quantum Dot Laser Considering Phonon Bottleneck, Carrier Relaxation and Homogeneous Broadening. *IEEE 2009 3rd ICTON Mediterranean Winter Conference*, 10.1109/ictonmw.2009.5385601 (2009).

[58] Levinshtein M (1996). Hand book series on semiconductor parameters. World Scientific.

